# RNase R, a New Virulence Determinant of *Streptococcus pneumoniae*

**DOI:** 10.3390/microorganisms10020317

**Published:** 2022-01-29

**Authors:** Cátia Bárria, Dalila Mil-Homens, Sandra N. Pinto, Arsénio M. Fialho, Cecília M. Arraiano, Susana Domingues

**Affiliations:** 1 Instituto de Tecnologia Química e Biológica António Xavier, Universidade Nova de Lisboa, 2780-157 Oeiras, Portugal; 2 iBB—Institute for Bioengineering and Biosciences and i4HB—Institute for Health and Bioeconomy, Instituto Superior Técnico, Universidade de Lisboa, 1049-001 Lisboa, Portugal; dalilamil-homens@tecnico.ulisboa.pt (D.M.-H.); sandrapinto@tecnico.ulisboa.pt (S.N.P.); afialho@tecnico.ulisboa.pt (A.M.F.); 3 Department of Bioengineering, Instituto Superior Técnico, Universidade de Lisboa, 1049-001 Lisboa, Portugal

**Keywords:** RNase, ribonuclease, *Galleria mellonella*, *Streptococcus pneumoniae*, virulence, pathogenicity, oxidative stress, hemocytes, NanA

## Abstract

Pneumococcal infections have increasingly high mortality rates despite the availability of vaccines and antibiotics. Therefore, the identification of new virulence determinants and the understanding of the molecular mechanisms behind pathogenesis have become of paramount importance in the search of new targets for drug development. The exoribonuclease RNase R has been involved in virulence in a growing number of pathogens. In this work, we used *Galleria mellonella* as an infection model to demonstrate that the presence of RNase R increases the pneumococcus virulence. Larvae infected with the RNase R mutant show an increased expression level of antimicrobial peptides. Furthermore, they have a lower bacterial load in the hemolymph in the later stages of infection, leading to a higher survival rate of the larvae. Interestingly, pneumococci expressing RNase R show a sudden drop in bacterial numbers immediately after infection, resembling the eclipse phase observed after intravenous inoculation in mice. Concomitantly, we observed a lower number of mutant bacteria inside larval hemocytes and a higher susceptibility to oxidative stress when compared to the wild type. Together, our results indicate that RNase R is involved in the ability of pneumococci to evade the host immune response, probably by interfering with internalization and/or replication inside the larval hemocytes.

## 1. Introduction

*Streptococcus pneumoniae* is an opportunistic pathogen that can be usually found as a harmless commensal of the human upper respiratory tract. Although this bacterium can colonize the nasopharynx of healthy individuals in an asymptomatic manner, propagation of pneumococcal cells beyond its niche along the nasal epithelium can lead to invasive diseases (reviewed in [1]). Dissemination either by aspiration, bacteremia, or local spread can result in infections, such as pneumonia, meningitis, otitis media, and septicemia. In fact, in 2017, *S. pneumoniae* was included by the World Health Organization (WHO) as one of the 12 priority pathogens. The switch that makes this bacterium change from colonizer to pathogen is triggered by the opportunity to invade the bloodstream, tissues, or organs of the host [2]. Translocation from the nasopharynx to deeper tissues exposes *S. pneumoniae* to different environmental niches, which require a rearrangement of the bacterial cell. Ribonucleases (RNases) are the enzymes which perform RNA maturation, degradation, and quality control and therefore they have a determinant role in the final expression levels of every transcript in the cell. Additionally, the control of the RNA level allows a faster response to sudden environmental changes [3]. In spite of their great importance, not much is known about pneumococcal ribonucleases. RNase Y and PNPase were recently shown to affect *S. pneumoniae* virulence in a murine model [4]. The transcriptional landscape of the respective mutants indicated a global effect of RNase Y on pneumococcal physiology and an implication of PNPase on *S. pneumoniae* virulence control, likely through sRNA regulation [4]. In agreement with this, PNPase was shown to interact with several noncoding RNAs in a RNA–protein complexome study [5]. In the same study, RNase YhaM was shown to promote stabilization of noncoding RNAs involved in pneumococcal competence [5]. We have recently demonstrated that RNase R affects translation and consequently impacts protein synthesis in *S. pneumoniae* [6]. Appropriate translation is essential for the modulation of gene expression needed to cope with environmental changes, such as the conditions that cause infection, which trigger the expression of virulence genes. A comprehensive understanding of the virulence mechanisms is of extreme importance for the development of new strategies to combat this bacterium. RNase R is important for virulence in several pathogens being upregulated in stress conditions. RNase R belongs to the RNase II/RNB family of enzymes, which are present in all domains of life [7,8,9]. This enzyme degrades RNA in the 3′–5′ direction in a processive and sequence-independent manner, being the only 3′–5′ exoribonuclease able to degrade highly structured RNAs [7]. RNase R is the only member of the RNB family present in *S. pneumoniae*, suggesting a major role in this microorganism. Therefore, we wanted to test if the absence of RNase R could compromise *S. pneumoniae*’s ability to cause infection.

Most of the studies on *S. pneumoniae* interaction with the host have been carried out in mammals (reviewed in [10]). However concerns related to the animal use, handle, and bioethics have led to the “three Rs” principle (replacement, reduction, and refinement) development [11]. With this in mind, alternative infection model organisms have been investigated, such as the larvae of the wax moth *Galleria mellonella*. This has been proven to be an effective model host for infection studies in several microorganisms ([12,13,14,15] reviewed by [16,17]), including *S. pneumoniae* [18]. Additionally, larvae can be reared at temperatures ranging from 20 °C to 30 °C and the infection studies can be conducted between 15 °C to above 37 °C, which enables experiments that attempt to mimic a human environment [19,20]. The innate immune system of *G. mellonella* involves cellular and humoral immune responses. The cellular response is mediated by phagocytic cells, termed hemocytes, that are involved in phagocytosis, encapsulation, and clotting. Hemocytes are found within the hemolymph and function analogously to mammalian neutrophils in terms of their ability to phagocytose and kill pathogens, through the production of superoxides [21,22,23,24]. The humoral response is composed of soluble effector molecules which immobilize and/or kill the pathogen when released into the hemolymph. This response includes the induced expression of antimicrobial peptides, as well as several plasma proteins that serve as opsonins, complement-like proteins, and release of the phenol oxidase system that culminates in the synthesis of the dark pigment melanin ([25,26,27] reviewed by [16]).

In this work, we used *G. mellonella* to study the impact of the exoribonuclease RNase R in the pneumococcus virulence. We show that RNase R has an important role in the pathogenicity of *S. pneumonia* and present evidence indicating that this enzyme might have a role in host–microbe interaction. Our results suggest that RNase R might promote the pneumococcus ability to evade the immune response of the host, probably by interfering with the proliferation of pneumococci inside the larvae hemocytes.

## 2. Materials and Methods

### 2.1. Bacterial Strains, Plasmids, Insects, and Growth Conditions

All bacterial strains and plasmids used in this study are listed in Table S1 (Supplementary Materials). *S. pneumoniae* strains are isogenic derivatives of the JNR7/87 capsulated strain—TIGR4. *S. pneumoniae* strains were grown in Todd Hewitt medium supplemented with 0.5% yeast extract (THY) at 37 °C without aeration or in THY agar medium supplemented with 5% sheep blood (Thermo Fisher Scientific, Waltham, MA, USA) at 37 °C in a 5% CO_2_ atmosphere. When required, growth medium was supplemented with 3 μg/mL chloramphenicol (Cm) or 1 μg/mL erythromycin (Ery).

*G. mellonella* larvae were reared on their natural food, beeswax, and pollen grains at 25 °C in darkness prior to use. Larvae weighing 225 ± 25 mg were used [19].

### 2.2. Flow Cytometry Analysis

Cellular growth was evaluated using S3e cell sorting equipment (Bio-Rad, Hercules, CA, USA). Overnight cultures of *S. pneumoniae* TIGR4 wild type and *rnr* mutant strain were diluted in prewarmed THY to a final OD_600_ of 0.05 and incubated at 37 °C. At OD_600_ ≈ 0.3, a volume of 15 mL of culture was centrifugated at 6000× *g* for 10 min. The cellular pellet was resuspended in sterile phosphate-buffered saline (PBS) with propidium iodide (PI) (Thermo Fisher Scientific, Waltham, MA, USA) at a final concentration of 10 μg/mL and incubated for 10 min in dark. Cells were centrifugated at 823× *g* for 10 min and subsequently resuspended in 1 mL sterile PBS. A total of 20 μL of the cell resuspension were transferred into a flow cytometry tube containing 1 mL of sterile PBS and analyzed in the flow cytometry equipment. Fluorescence was detected using FL1 and FL2 filters. Live cells and death cells from old cultures were used as controls to set up gating.

### 2.3. Galleria mellonella Killing Assay

For *G. mellonella* infection, overnight cultures of *S. pneumoniae* TIGR4 wild type and derivatives were diluted in prewarmed THY to a final OD_600_ of 0.05 and incubated at 37 °C. At OD_600_ ≈ 0.3, an appropriate volume was collected to ensure that the same number of cells was used for all the strains. Cells were then harvested by centrifugation and resuspended in sterile PBS in a series of 10-fold serial dilutions corresponding to the number of 5 × 10^6^ bacterial cells per volume of injection. A micrometer was adapted to control the volume of a microsyringe, and 5 μL aliquots of each dilution were injected into *G. mellonella*, via the hindmost left proleg, which had been previously surface-sterilized with 70% ethanol [15]. Control larvae were injected with the same volume of sterile PBS. Following injection, larvae were placed in glass Petri dishes and incubated in the dark at 37 °C for 3 days. For each condition, the survival and appearance of 10 larvae were followed at 24 h intervals. Larvae were considered dead when they displayed no movement in response to touch. At least three independent experiments were performed for each condition.

### 2.4. RNA Extraction

For extraction of RNA from *G. mellonella*, sets of 20 larvae were infected with *S. pneumoniae* strains at a concentration of 5 × 10^6^ bacterial cells per larvae, as previously described for the killing assays. At 1, 6, 12, and 18 h after injection, three living larvae per set were cryopreserved, sliced, and homogenized in 1 mL of TRIzol reagent (Sigma-Aldrich, St. Louis, MO, USA). Whole-animal RNA was extracted according to the manufacturer’s protocol. After extraction, RNA was treated with an RNase-free DNase set (Qiagen, Germantown, MD, USA). The purified RNA was quantified spectrophotometrically (NanoDrop ND-1000, Wilmington, DE, USA).

For extraction of RNA from *S. pneumoniae*, overnight cultures of *S. pneumoniae* TIGR4 wild type and derivatives were diluted in prewarmed THY to a final OD_600_ of 0.1 and incubated at 37 °C. At OD_600_ ≈ 0.3, 20 mL of culture was collected, mixed with 1 volume of stop solution (10 mM Tris pH 7.2, 25 mM NaNO_3_, 5 mM MgCl2, 500 µg/mL Cm), and harvested by centrifugation (10 min, 6000× *g*, 4 °C). Total RNA was extracted using TRIzol reagent (Ambion, Austin, TX, USA) essentially as described by the manufacturer, with some previously described modifications [6]. RNA integrity was evaluated by gel electrophoresis and RNA concentration was estimated using a Nanodrop 1000 machine (Nanodrop Technologies, Wilmington, DE, USA).

In both cases, RNA extraction was performed from at least three independent biological samples.

### 2.5. Quantitative Real-Time PCR

Reverse transcription coupled to quantitative PCR (RT-qPCR) was performed with a Real Time Thermal Cycler qTower (Analytik Jena, Jena, Germany) system using a SensiFast SYBR kit (Bioline, London, UK) according to the supplier’s instructions. cDNA was synthesized from 1 μg of purified RNA with the SensiFast cDNA synthesis kit (Bioline, London, UK). The primers used are listed in Table S2 (Supplementary Materials). All samples were analyzed at least in triplicate, from three independent biological samples. Expression of *Galleria* genes was normalized to the mRNA amount of the housekeeping gene actin, while expression of *S. pneumoniae* genes was normalized using the pneumococcal *recP* mRNA. Relative quantification of gene expression was calculated by using the ΔΔCt (Ct is threshold cycle) method [28].

### 2.6. Extraction of Larval Hemolymph

To determine the number of viable bacteria in the hemocoel during the course of infection at 1, 6, and 18 h after injection, the hemolymph of infected larvae was collected as previously described [15]. Briefly, three living larvae were anesthetized on ice and surface-sterilized with ethanol. The larvae were bled, and the outflowing hemolymph was immediately transferred into a sterile microtube containing a few crystals of phenylthiourea to prevent melanization [29]. Bacteria were enumerated by colony forming unit (CFU) counting after incubation at 37 °C for 24 h, and results are presented as the number of CFU per milliliter of hemolymph.

### 2.7. G. mellonella Hemocytes In Vitro Culture

For *G. mellonella* hemocytes isolation, hemolymph was extracted from last-instar larvae, previously sanitized with ethanol, by puncturing the larval abdomen with a sterile needle [30]. Hemolymph was directly collected into a microcentrifuge tube containing 100 μL of anticoagulant buffer (98 mM NaOH, 145 mM NaCl, 17 mM EDTA, and 41 mM citric acid; pH 4.5) (Sigma-Aldrich, St. Louis, MO, USA) in a 1:1 proportion. The hemolymph was centrifuged at 250× *g* for 10 min at 4 °C and the hemocytes recovered were washed twice with PBS, and centrifuged at 250× *g* for 5 min at 4 °C. Finally, hemocytes were gently suspended in 1 mL of Grace’s insect medium (GIM) (Gibco, Thermo Fisher Scientific, Waltham, MA, USA) supplemented with 10% (vol/vol) fetal bovine serum (Lonza, Basel, Swiss), 1% (wt/vol) glutamine, and 1% (wt/vol) antibiotic/antimycotic solution (10,000 units of penicillin G, 10 mg of streptomycin, 25 mg/L amphotericin B) (Gibco, Thermo Fisher Scientific, Waltham, MA, USA). Hemocytes were enumerated with a hemocytometer and incubated at 26 °C in 24-well plates containing glass coverslips, at a concentration of 2 × 10^5^ cell/well. Monolayers of primary *Galleria* hemocytes were used for ex vivo experiments the next day.

### 2.8. Gentamicin Protection Assay of Hemocytes and CLSM

To evaluate the intracellular bacterial load of ex vivo hemocytes during *S. pneumoniae* infection, a protocol based on the gentamicin protection assay followed by visualization with confocal laser scanning microscopy (CLSM) and two-photon microscopy was performed in accordance with previous descriptions [31]. Prior to infection with bacteria, *G. mellonella* hemocyte monolayers were washed with PBS, and medium was replaced with GIM without supplements. *S. pneumoniae* strains were grown as described above, and the appropriate volume was collected to obtain 5 × 10^4^ bacteria in each well. After 1 h of infection at 37 °C, the hemocytes were carefully washed twice with PBS, followed by the addition of GIM containing 100 mg/L of gentamicin to kill extracellular bacteria. After 1 h of incubation, medium was replaced with GIM containing 10 mg/L of gentamicin. After each infection time points, the wells were washed with PBS, and the glass coverslips were fixed with 3.7% (wt/vol) paraformaldehyde for at least 20 min at 4 °C. Then, samples were quenched with 50 mM NH_4_Cl (Sigma-Aldrich, St. Louis, MO, USA) for 10 min, immersed in 0.2% (vol/vol) Triton X-100 (Sigma-Aldrich, St. Louis, MO, USA) for 5 min, and saturated with 5% (wt/vol) bovine serum albumin (BSA) (Sigma-Aldrich, St. Louis, MO, USA) for 30 min. Between each step, the samples were washed three times with PBS. The bacterial immunostaining was then performed using a monoclonal antibody against *S. pneumoniae* (1:50) in PBS with 5% BSA (Thermo Fisher Scientific, Waltham, MA, USA), followed by the addition of a secondary polyclonal goat anti-rabbit serum coupled to Alexa 488 (1:500) (Santa Cruz Biotechnology, Dallas, TX, USA). Hemocytes were stained using wheat germ agglutinin (WGA) conjugated with Alexa 633 (1:200) (Thermo Fisher Scientific, Waltham, MA, USA). Hoechst 33342 fluorescent dye (Thermo Fisher Scientific, Waltham, MA, USA) was used for nuclear staining (1:500). Finally, coverslips were mounted in Vectashield (Vector Laboratories, Burlingame, CA, USA). All samples were examined on a Leica TCS SP5 (Leica Microsystems CMS GmbH, Wetzlar, Germany) inverted microscope (model no. DMI6000) with a 63× water apochromatic objective. The Hoechst dye was visualized using two-photon excitation microscopy (Ti: sapphire laser, Spectra-Physics Mai Tai BB, 710–990 nM, 100 fs, 82 MHz).

Typically, images were collected with 512 × 512 pixels and at a scan rate of 100 Hz per frame.

### 2.9. Oxidative Stress Assay

Mid-log phase cultures of TIGR4 wild type and derivatives (OD at 600 nM ~ 0.3–0.4) were challenged with 5 mM of hydrogen peroxide and incubated at 37 °C. Aliquots were collected after 15 min and 30 min, serially diluted in sterile PBS, and plated on THY supplemented with 5% sheep blood for CFU enumeration. The results were obtained from at least three independent assays and were normalized to the number of cells obtained with the corresponding untreated bacteria.

### 2.10. Statistical Analysis

Data are expressed as mean values of a minimum of three independent experiments ± standard deviations (SD). Statistical analysis was carried out using Graphpad 8.0 (San Diego, CA, USA). All *p* values were calculated using Student’s *t*-test and a value of <0.05 was considered statistically significant.

## 3. Results

### 3.1. Effect of Pneumococcal RNase R on Galleria mellonella Infection

It was our main goal to evaluate the virulence potential of the *S. pneumoniae* RNase R mutant strain (Δ*rnr*). In virulence studies, inoculation of the host with the same number of viable cells is essential. In order to evaluate its planktonic growth, we used flow cytometry, allowing detection of live or death cells in the bacterial culture through the use of propidium iodide (PI) dye. The wild type and *rnr* mutant strain were grown in identical conditions, and the profile and number of live cells within the cultures were compared. The number of live cells at the same OD_600_ was identical for both strains (Figure S1A, Supplementary Materials). The proportion of live and death cells were identical in the wild type and the Δ*rnr* strain, as judged by the PI ability to penetrate only the damaged, permeable membranes of nonviable cells. Indeed, the PI signal is detected in a low and identical number of cells for both wild type and RNase R mutant strain (Figure S1B, Supplementary Materials). Our results indicate that deletion of RNase R has no impact in the number of cells during exponential growth.

To evaluate the virulence potential of the strain deficient in RNase R, we compared the survival rates of *G. mellonella* infected with the wild type strain, the Δ*rnr* strain, and the Δ*rnr* strain complemented with RNase R (Δ*rnr*+R). We started by evaluating the pathogenicity level of the wild type strain by determining the dose of bacteria lethal for 50% of *G. mellonella*. For this, serial dilutions of the wild type were used to inoculate *G. mellonella*. Larvae survival and melanization was observed every 24 h until 72 h after inoculation. After 24 h of incubation, all the larvae infected with the wild type at 10^7^ bacteria/larva were dead and presented strong melanization (Figure S2, Supplementary Materials). When 10^6^ bacteria/larva were used, 50% of larvae died and presented less melanization, while for doses below 10^6^ bacteria/larva, all larvae survived even after 72 h of incubation. Thus, the lethal dose of the wild type bacteria necessary to kill 50% of larvae (LD_50_) is 5 × 10^6^ bacterial cells/larva, which is in agreement with the results previously obtained [18].

**Figure 1:**
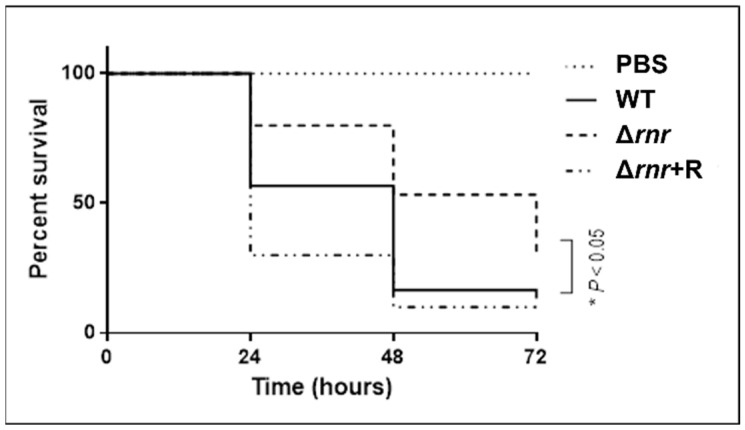
Survival of *G. mellonella* larvae after inoculation with *S. pneumoniae* wild type and RNase R mutant derivatives. Kaplan–Meier survival curves representing larvae infected with 5 × 10^6^ bacteria/larva. Larvae were infected with *S. pneumoniae* wild type (WT), *rnr* mutant (∆*rnr*), ∆*rnr* expressing RNase R in trans (∆*rnr*+R), and phosphate-buffered saline (PBS) as a control for 72 h. The median survival time after infection with ∆*rnr* is 72 h, whereas with the wild type is 48 h, and only 24 h after infection with ∆*rnr*+R. The results represent three independent experiments.

To compare the survival rate of the larvae infected with the wild type and mutant strains, 5 × 10^6^ bacteria/larva (LD_50_) were thus used to inoculate *G. mellonella*. Survival of the larvae was evaluated as described above. As expected, the effects of infection by the wild type strain were rapidly seen after the first 24 h of inoculation, with a reduction of about 50% of the initial larval population (Figure 1). After 72 h of incubation, only about 10% of the larvae survived. In contrast, deletion of RNase R increased, by 30%, the larval survival rate in the course of infection (Figure 1). Complementation of the mutant strain with RNase R expression in trans seemed even more lethal than the wild type after 24 h, but the wild type survival rates were restored 72 h after infection (Figure 1).

These results demonstrate that the virulence potential of *S. pneumoniae* is attenuated in the absence of RNase R, establishing the importance of this ribonuclease in the capacity of this bacterium to cause infection.

### 3.2. RNase R Interference with G. mellonella Immune Response

**Figure 2:**
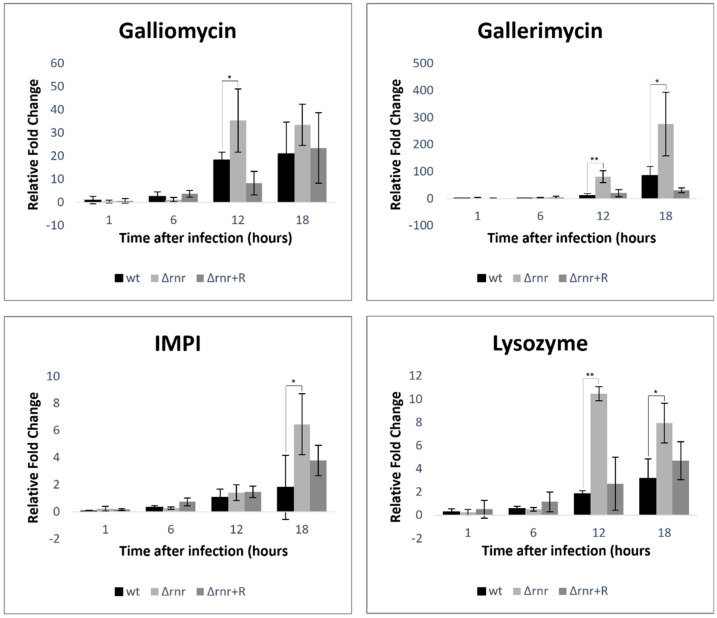
Immunogenic response of *G. mellonella* during infection with *S. pneumoniae* TIGR4 and derivatives. Expression levels of the immune-responsive genes of *G. mellonella* were measured at 1, 6, 12, and 18 h postinfection with 5 × 10^6^ bacteria/larva of the *S. pneumoniae* TIGR4 wild type (wt), RNase R mutant (∆*rnr*), ∆*rnr* expressing RNase R *in trans* (∆*rnr*+R), and PBS (as a control). The transcriptional levels of gallerimycin, galliomycin, IMPI, and lysozyme were determined by quantitative RT-PCR analysis and compared between the larvae infected with the wild type (wt) and mutant strains (∆*rnr* and ∆*rnr*+R). Results were normalized to the expression of the housekeeping gene actin and are shown relative to the corresponding expression levels in noninfected larvae injected with PBS. These data are representative of three independent experiments (* *p* < 0.05; ** *p* < 0.01).

Host defense peptides are a crucial part of insect innate immunity against invading pathogens, showing broad-spectrum microbicidal activity [32]. Therefore, antimicrobial peptides induction by *G. mellonella* are expected upon *S. pneumoniae* infection, as previously suggested [18]. To test this, larvae were infected with the wild type strain, and the gene expression of four selected antimicrobial peptides was monitored at 1, 6, 12, and 18 h postinfection. The selected genes coded for the cysteine-rich antifungal peptide gallerimycin [33], a defensin called galliomycin [16], lysozyme [33], and an inducible metalloproteinase inhibitor (IMPI) [33]. Gene expression was determined by quantitative RT-PCR analysis of the total RNA extracted for each postinfection time point. To study the impact of RNase R, the expression of these peptides was also measured in the Δ*rnr* and complemented (Δ*rnr*+R) strains. As expected, our results showed an increased expression of the immune-related peptides after infection with the wild type compared to the control larvae injected with PBS (Figure 2). However, the expression levels of all the tested immune-related peptides increased several fold within the larvae injected with the RNase R mutant strain, and the induction was generally more pronounced for the later times after injection. In all the cases, injection with the complemented strain partially reverted to the wild type values, clearly indicating a role for RNase R in the activation of the insect immune response. This fact might be related to the lower virulence potential of the strain lacking RNase R.

### 3.3. RNase R Impact on the Bacterial Load in Hemolymph

**Figure 3:**
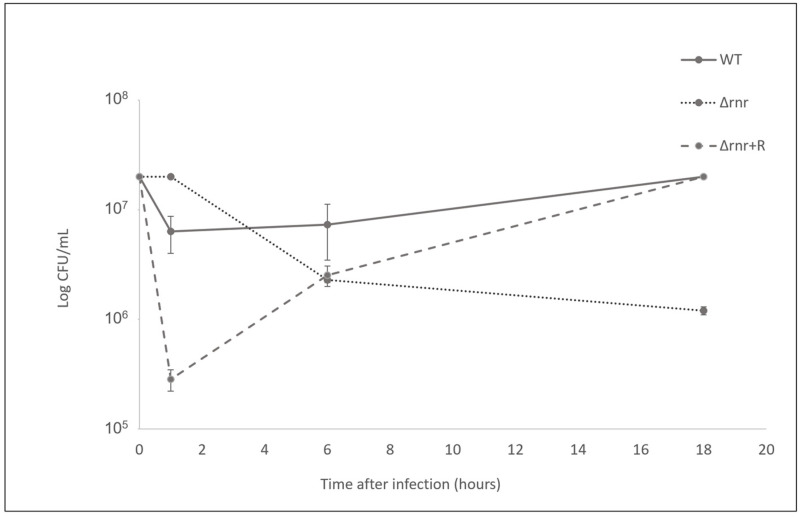
Hemolymph bacterial load in the course of *S. pneumoniae* infection. The viable bacterial load was determined in the hemolymph extracted from larvae infected with 5 × 10^6^ bacteria/larvae of the *S. pneumoniae* wild type (wt), RNase R mutant (∆*rnr*), and complemented (∆*rnr*+R) strains during a period within the time course of infection. Results (number of colony forming units (CFU) per mL of hemolymph obtained at 1, 6, and 18 h postinfection) represent means of at least three independent experiments.

High mortality is associated with bacterial proliferation within the host, while low mortality is associated with bacterial clearance [18]. In order to evaluate the proliferation of *S. pneumoniae* within the insect hemocoel, we determined the viable bacterial load within the larvae hemolymph at the course of infection. At 1, 6, and 18 h after injection of the larvae with *S. pneumoniae* wild type, or RNase R mutant derivatives, hemolymph from three living larvae was collected and pooled, and the number of CFU was determined (Figure 3). Surprisingly, after 1 h, the counts of the wild type dropped down, and a sharp decrease was also observed for the strain complemented with RNase R, whereas the values of the RNase R mutant remained constant. Nonetheless, at 6 h postinfection, both the wild type and the complemented strain started to recover and increasing numbers of bacteria could be detected in larval hemolymph 18 h postinfection.

On the contrary, the RNase R mutant suffered a severe decrease 6 h after infection and could not recover. At 18 h postinfection, the bacterial load in hemolymph was significantly lower for the RNase R mutant than for the wild type strain, which is indicative of a role for RNase R in the ability of *S. pneumoniae* to proliferate inside the host. The lower proliferation of the ∆*rnr* inside the larvae is in agreement with the higher survival of the larvae infected with this strain.

### 3.4. RNase R Affects the Number of Intracellular Bacteria in Hemocyte Cultures

**Figure 4:**
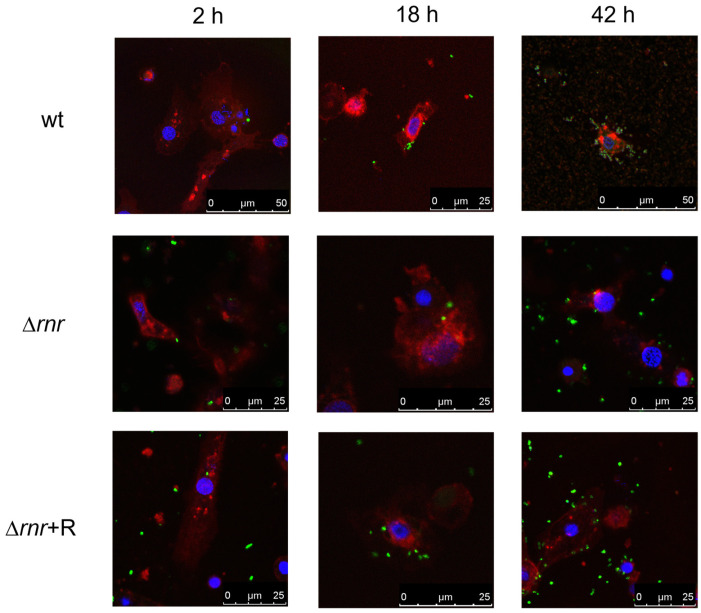
*G. mellonella* hemocytes as ex vivo infection model for *S. pneumoniae* strains. Fluorescence confocal microscopy images of the primary hemocytes cell cultures infected with the *S. pneumoniae* TIGR4 wild type (wt), RNase R mutant (∆*rnr*), and ∆*rnr* expressing RNase R in trans (∆*rnr*+R) strains at 2, 18, and 42 h postinfection. Hemocyte membranes were labeled with WGA conjugated with Alexa 633 (red), while bacteria were stained with *S. pneumoniae* monoclonal antibody and Alexa 488 (green), and Hoechst dye was used for staining the cell’s nucleus (blue). Experiments were repeated at least three times, and one representative experiment of the overlay between the three dyes is shown.

Although the pneumococcus has been considered a typical extracellular pathogen, recent reports have highlighted the importance of intracellular replication for bacterial survival and pathogenesis [2]. Therefore, to further study the effect of *S. pneumoniae* RNase R deletion in the host–pathogen interaction in the *G. mellonella* model, the insect hemocytes were extracted from larval hemolymph and used as ex vivo model. The primary cell cultures of hemocytes were infected with *S. pneumoniae* TIGR4 wild type, ∆*rnr*, and ∆*rnr*+R strains, and the bacterial amount within hemocytes was assessed by gentamicin protection assay followed by visualization with CLSM and two-photon excitation microscopy up to 42 h of infection. After 2 h, no significant differences were observed (Figure 4). However, during the course of the experiment, differences between the wild type and the mutant became apparent; at 18 h postinfection, the wild type presented a higher number of internalized bacteria. This result is corroborated by the complemented strain (Figure 4). Consistent with this result, we found, at 42 h postinfection, higher intracellular bacterial division in hemocytes infected with the wild type strain compared to the mutant strain. At this point of the infection, the wild type phenotype was partially restored. Despite that the initial cellular uptake of the wild type and the *rnr* mutant strains had similar levels, in the course of infection, the wild type strain seemed to be more adapted to proliferate inside the hemocytes.

### 3.5. RNase R Impact in the Pneumococcus Oxidative Response

**Figure 5:**
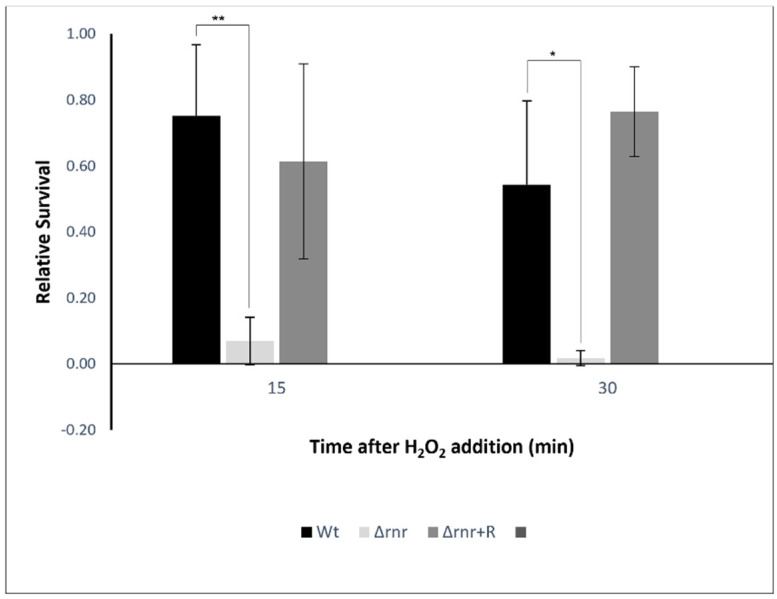
Hydrogen peroxide susceptibility of *S. pneumoniae* wild type and ∆*rnr* mutant derivatives. Exponential cultures were challenged for 15 min and 30 min with 5 mM H_2_O_2_. Viable cells were extrapolated by plating the appropriate dilutions on Todd Hewit medium supplemented with 0.5% Yeast extract (THY) agar plates supplemented with 5% sheep blood. The results were normalized and are presented relative to the number of viable cells obtained for the same culture before stress induction. The results represent an average of at least three independent experiments (* *p* < 0.05; ** *p* < 0.01).

Microbial killing by phagocytosis is mainly attributed to the oxidative burst produced by macrophages and neutrophils [34], and the similarities between the oxidative burst pathways of insect hemocytes and mammalian neutrophils are well known [21,35]. Production of reactive oxygen species (ROS), such as the superoxide and hydrogen peroxide (H_2_O_2_), has been observed in *G. mellonella* [21,36]. The lower number of intracellular bacteria observed in the ∆*rnr* could be due to a decreased resistance to oxidative stress of this strain. Thus, we compared the survival rate between the RNase R mutant and the wild type, when cultured in the presence of 5 mM H_2_O_2_. When compared to the wild type, our results show a significant decrease of Δ*rnr* growth as soon as 15 min after the stress challenge (Figure 5). At 15 min post-exposure, the wild type decreased 25%, whereas the mutant showed a reduction of 93%. As expected, the survival of both strains was lower after 30 min. These results show that, compared to the wild type, the Δ*rnr* is significantly more susceptible to hydrogen peroxide. Susceptibility of the complemented strain was comparable to that of the wild type, clearly indicating a role for RNase R in the ability of the pneumococcus to cope with oxidative stress. This might be, at least in part, the cause for the reduced internalized bacteria.

### 3.6. Transcriptional Expression of NanA Is Affected by RNase R

**Figure 6:**
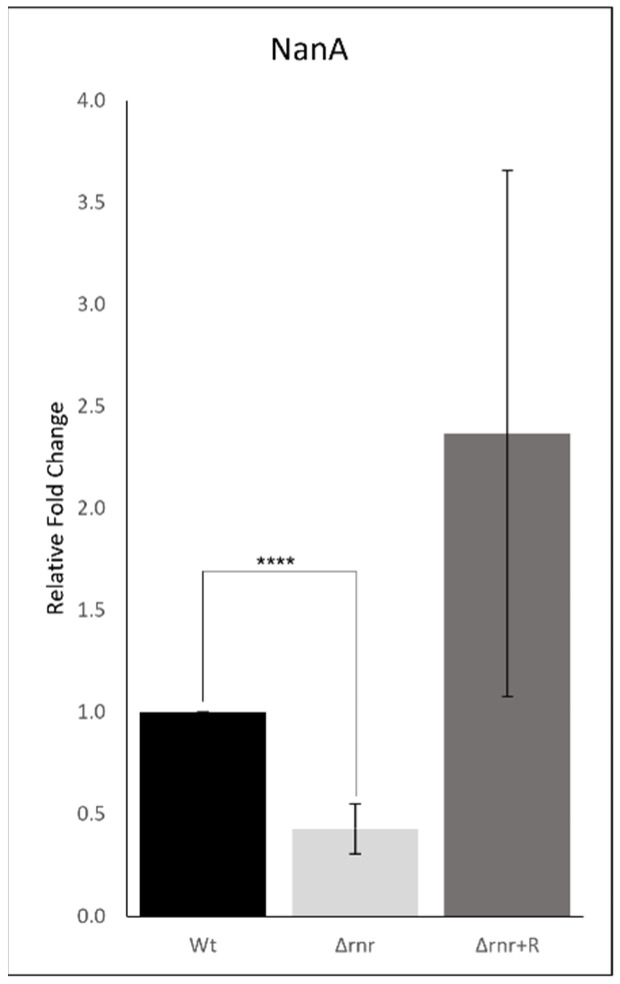
Comparative expression of NanA message by *S. pneumoniae* wild type and derivatives. The transcriptional levels of NanA were determined by quantitative RT-PCR analysis in exponential growing pneumococcal cultures (wild type—wt; Δ*rnr*; Δ*rnr*+R). Results were normalized to the expression of the pneumococcal housekeeping gene *recP* and are shown relative to the expression levels in the wild type strain. These data are representative of at least three independent experiments (**** *p* < 0.0001).

A set of virulence factors has been shown to be involved in the ability of the pneumococcus to internalize and survive intracellularly [2]. Therefore, we thought that the lower level of Δ*rnr* mutant inside hemocytes could also be due to interference of RNase R in the expression of these genes. The expression levels of some of these virulence factors were evaluated by RT-qPCR and compared between the wild type and the mutant derivatives. We analyzed the transcriptional levels of pneumolysin (Ply), pneumococcal surface protein A (PspA), the pilus-1 adhesin RrgA, pyruvate oxidase (SpxB), and neuraminidase A (NanA). No significant variations were observed on the levels of Ply, PspA, RrgA, or SpxB (Figure S3); however, we detected a lower amount of the NanA transcript in the Δ*rnr* strain (Figure 6).

Compared to the wild type, the expression level of this transcript was reduced by about 60%. NanA is an important adhesin that has been associated with pneumococcal internalization, and its decreased amount may well contribute to the lower number of mutant bacteria observed inside the hemocytes. A reversion to higher expression levels was observed in the complemented RNase R strain. However, in this strain, the NanA transcriptional levels were increased relative to the wild type amount, which might be due to the augmented level of plasmid-expressed RNase R [37]. Together, these results are indicative of a role for RNase R in the modulation of this virulence factor.

## 4. Discussion

Bacteria have developed a wide range of virulence mechanisms that are critical for successfully infect the host. A proper coordination of the bacterial cellular processes is essential for the progression of infection. RNase R is a stress response exoribonuclease, whose expression increases under several growth conditions [9,38,39,40,41]. Involvement of this enzyme in virulence has been described in several pathogens [8] and its impact in protein synthesis and translation was recently shown in *S. pneumoniae* [6]. Therefore, in this work we investigated whether RNase R would have a role in the pathogenicity of the pneumococcus. We show that the absence of RNase R leads to a decrease in *S. pneumoniae* virulence. Using *G. mellonella* as an infection model, our results demonstrate that 24 h after inoculating *G. mellonella* larvae with the wild type, only half of the larvae survived, while after infection with the Δ*rnr* strain, around 75% of the larvae survived during the same period. These differences were accentuated after 3 days, with a survival rate of only 10% after infection with the wild type, compared to 30% after inoculation with Δ*rnr*. Interestingly, infection with the strain expressing RNase R in trans even diminishes the survival rate. This is indicative of a direct role of RNase R in the pneumococcus pathogenicity, as, in this strain, RNase R levels are higher than in the wild type [37].

The Toll pathway, a crucial signaling cascade of the insect innate immune system, is activated by fungi and Gram-positive bacteria, leading to the synthesis of antimicrobial peptides [16,42]. *G. mellonella* contains a notable antimicrobial peptide arsenal that is released into the hemolymph, where it attacks elements of the bacterial or fungal cell wall [25]. We found that infection with the wild type strain triggered the expression of the four antimicrobial peptides tested here (gallerimycin, galliomycin, IMPI, and lysozyme). Remarkably, higher expression levels of all the corresponding transcripts were detected when the larvae were infected with the deleted RNase R strain, especially for the later infection times. Hence, the absence of RNase R seems to induce a stronger humoral response by the larvae. This could be due to a higher immunogenicity of this strain but may also result from evasion of the immune response by the wild type, whereas the mutant would have lost this skill.

Although the pneumococcus is considered a typical extracellular pathogen, its ability to proliferate within the host cells has recently been recognized [2,43,44]. Efficient clearance of pneumococci by macrophages and neutrophils was observed following intravenous inoculation of mice. However, occasional intracellular proliferation of these sequestered bacteria provided a reservoir for subsequent dissemination of pneumococci into the bloodstream. The internalized bacteria evade clearance, undergo replication, then cause macrophage lysis and blood dissemination [43]. Endocytosis-mediated internalization into cardiomyocytes followed by pneumococci replication within intracellular vacuoles was also reported [44]. In the lungs, the uptaken pneumococci were proposed to persist intracellularly within dendritic cells and macrophages [45]. The accentuated decrease of the wild type and complemented mutant in the larvae hemolymph 1 h after infection strongly resembles the eclipse phase, whereby pneumococci numbers rapidly decrease in the bloodstream after intravenous inoculation [43]. Indeed, after the initial drop in bacterial numbers, a recovery of both strains was observed with the respective load increasing until at least 18 h postinfection, resembling pneumococci blood dissemination after the eclipse. By contrast, the bacterial load of the strain lacking RNase R, although higher 1 h after infection, constantly decreased, and the strain could not recover. The other branch of innate immunity is cellular defense, mediated by *G. mellonella* hemocytes. They have similarities with human macrophages and neutrophils, being able to phagocytose pathogens [16,46]. In agreement with this, we detected bacterial foci inside larval hemocytes, but in the absence of RNase R, a reduced number of foci was observed when compared to the wild type. Our data on hemocytes infection suggests that proliferation of the mutant bacteria inside the hemocytes might be deficient. In support of this result, the susceptibility to oxidative stress was strongly increased in the Δ*rnr*, which might compromise the ability of the deleted strain to cope with the oxidative burst inside hemocytes. RNase R is a stress-responsive exoribonuclease, but to our knowledge, this is the first time that it has been implicated in resistance to oxidative stress. However, the transcriptional levels of SpxB, one of the virulence factors particularly important for ensuring pneumococcal survival under oxidative stress, were unchanged in the absence of RNase R (Figure S3). The role of RNase R in the resistance to oxidative stress remains to be elucidated.

The RNase R mutant also showed reduced expression levels of NanA, a virulence factor reported to be implicated in pneumococcal invasion. NanA is sialidase that releases sugars from host glycoproteins, exposing host receptors for pneumococcal adhesion [47,48]. It is known to be involved in sialic acid-mediated host cell binding and invasion [48], and a *nanA* deletion mutant showed reduction of the number of foci in the metallophilic macrophages [43]. Moreover, the presence of sialic acid in the prothoracic glands of *G. mellonella* larvae [49] might be supportive for a role of NanA in *G. mellonella* infection by pneumococci. Although hemocytes infection appeared similar in the first hours after infection with the three strains, the reduced level of NanA in the deleted RNase R strain might also compromise the pneumococci uptake by hemocytes, presumably also accounting for the lower Δ*rnr* foci observed inside the larvae hemocytes. In agreement with this, previous works on *Escherichia coli*, *Shigella flexneri,* and *Campylobacter jejuni* have shown that Δ*rnr* mutant strains were less invasive [50,51].

Together, our results are consistent with engulfment of pneumococci by the larval hemocytes and are indicative of a role for RNase R in facilitating replication inside the hemocytes. This would also explain the higher expression of the antimicrobial peptides in the larvae infected with the Δ*rnr*, which would thereby fail to be “hidden” inside the hemocytes. Our results indicate that the strains containing RNase R can evade the insect immune response, while in the absence of this enzyme the innate immune system is able to cope with the infection. RNase R might thus have an important role in the establishment of invasive disease, probably by affecting pneumococci phagocytosis. It would be very interesting to pursue these studies, investigating the invasive infection in a mammal model of infection with the RNase R mutant, or study intracellular replication of Δ*rnr* pneumococci in an ex vivo porcine spleen, where the microanatomy is comparable to humans.

We present evidence showing that the RNase R might interfere with the pneumococcus interaction with the innate immune system of the host. The fact that the insect and human innate immune responses are very similar is suggestive of a role for RNase R in the eclipse phase previously observed in mice infections. Overall, this work highlights the importance of RNase R as a regulator of pneumococcus virulence.

## Data Availability

Not applicable.

## Supplementary Materials

The following are available online at https://www.mdpi.com/article/10.3390/microorganisms10020317/s1, Figure S1: Flow cytometry analysis of *S. pneumoniae* wild type and Δ*rnr* cultures at exponential growth, Figure S2: *G. mellonella* survival following infection with pneumococcal cells, Figure S3: Comparative expression of Ply, PspA, RrgA, and SpxB messages by *S. pneumoniae* wild type and derivatives, Table S1: Strains and plasmids used in this work, Table S2: Oligonucleotides used as primers in this work. References [52,53,54] are cited in the supplementary materials.
