# Supplementary material for: RNase R, a New Virulence Determinant of Streptococcus pneumoniae
Source: Microorganisms. 2022 Jan 29;10(2):317. doi: 10.3390/microorganisms10020317 (PMC8875335; doi:10.3390/microorganisms10020317)
Supplement: Supplementary file 1 [file microorganisms-10-00317-s001.zip › microorganisms-1534602-supplementary.pdf]

Figure S1

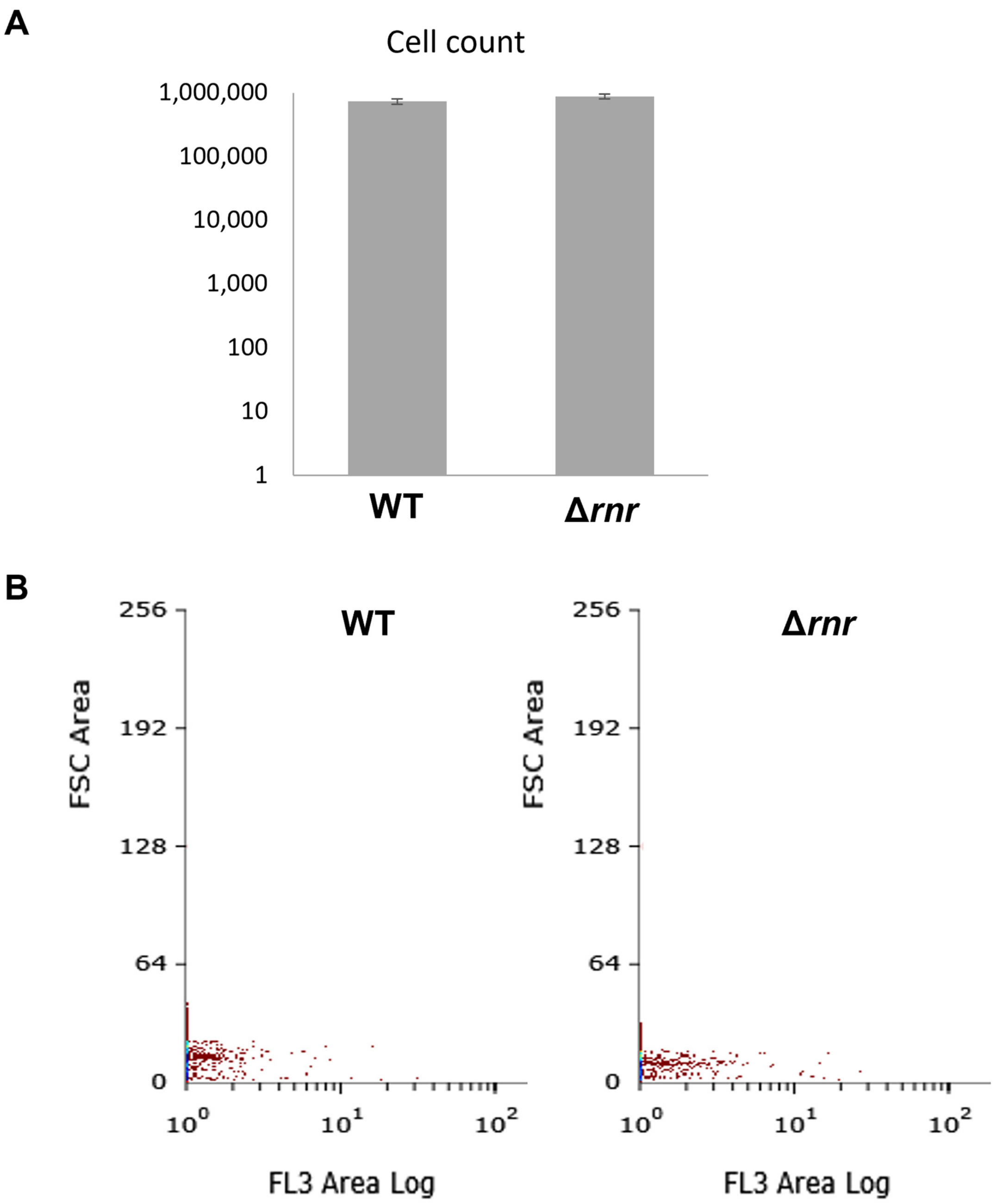

Figure S1. Flow cytometry analysis of *S. pneumoniae* wild type and Δ*rnr* cultures at exponential growth. Analysis of *S. pneumoniae* wild type (WT) and *rnr* mutant (Δ*rnr*) cells growing in liquid media. (**A**) Graphical representation of the number of live cells in each sample. (**B**) Detection of propidium iodide signal. For all the experiments a low flow was used, and the percentage of live/dead cells was determined in 10 sec gated events. Unstained cells and dead cells from old cultures were used as negative and positive control, respectively.

Figure S2

A

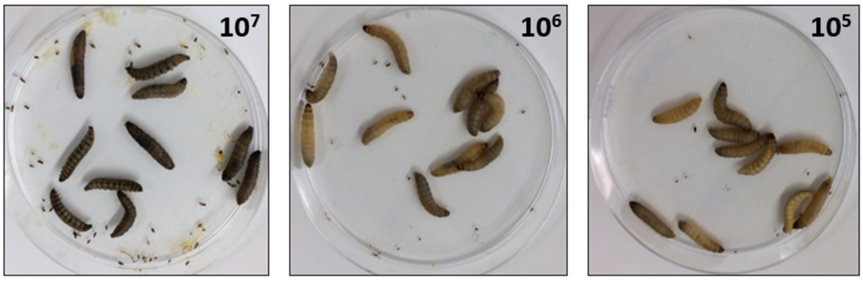

B

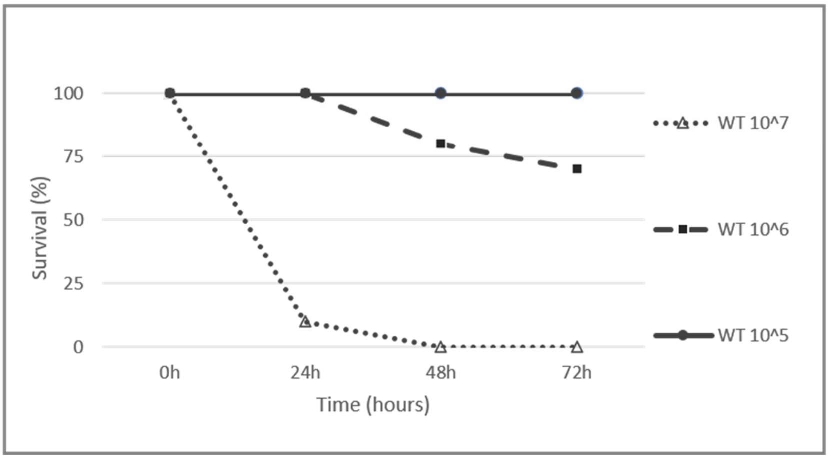

Figure S2. *G. mellonella* survival following infection with pneumococcal cells. Ten Larvae were inoculated with each serial dilution of *S. pneumoniae* wild type culture (number of bacterial cells is indicated on the top right corner of the corresponding photo, and on the right of the graph). Melanization (**A**) and Survival rate (**B**) of the larvae after 24 h of incubation at 37 °C are shown.

Figure S3

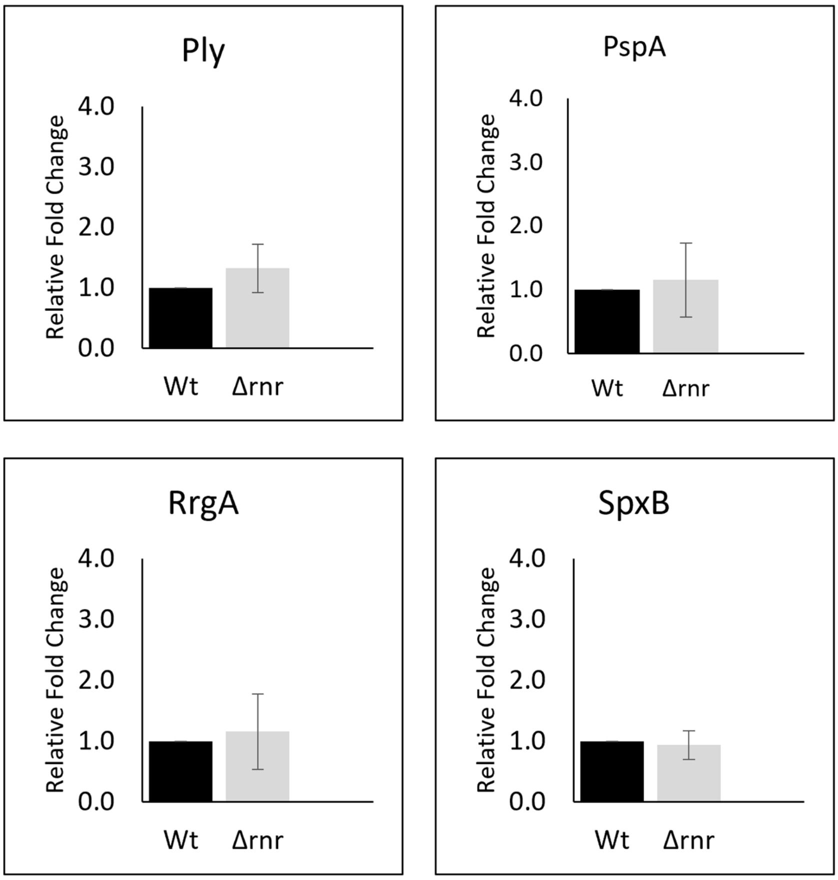

Figure S3. Comparative expression of Ply, PspA, RrgA and SpxB messages by *S. pneumoniae* wild type and derivatives. The transcriptional levels were determined by quantitative RT-PCR analysis in exponential growing pneumococcal cultures (wild type – wt; Δ*rnr*). Results were normalized to the expression of the pneumococcal housekeeping gene *recP* and are shown relative to the expression levels in the wild type strain. These data are representative of at least three independent experiments.

Table S1. Strains and plasmids used in this work.

Strain/Plasmid	Relevant characteristics	Reference
**Bacteria**		
S. pneumoniae		
JNR7/87 (TIGR4)		[1]
CMA607	TIGR4 carrying pIL253 (Ery^R)	[2]
CMA611	TIGR4 *rnr*⁻ (Δ*rnr*) (Cm^R)	[2,3]
CMA604	CMA611 carrying pIL253 (Ery^R) expressing RNase R (Δ*rnr*+R) (Cm^R)	[3]
CMA612	CMA611 carrying pIL253 (Ery^R)	[4]
**Plasmids**		
pIL253	pAMβ1 derivative (Ery^R)	[2,5]
pIL253-RNaseR	pIL253 carrying pneumococcal RNase R (Ery^R)	[3]

Ery^R: Erytromycin resistant; Cm^R: Cloramphenicol resistant.

Table S2. Oligonucleotides used as primers in this work.

Oligo name	Sequence 5′ to 3′	Reference
P1RT (gallerimycin)	CGCAATATCATTGGCCTTCT	[6]
P2RT (gallerimycin)	CCTGCAGTTAGCAATGCAC	[6]
P1RT (IMPI)	AGATGGCTATGCAAGGGATG	[6]
P2RT (IMPI)	AGGACCTGTGCAGCATTTCT	[6]
P1RT (lysozyme)	TCCCAACTCTTGACCGACGA	[6]
P2RT (lysozyme)	AGTGGTTGCGCCATCCATAC	[6]
P1RT (actin)	ATCCTCACCCTGAAGTACCC	[6]
P2RT (actin)	CCACACGCAGCTCATTGTA	[6]
P1RT (galliomycin)	TCGTATCGTCACCGCAAAATG	[7]
P2RT (galliomycin)	GCCGCAATGACCACCTTTATA	[7]
CBR007 (recP)	GCCAACTCAGGTCATCCAGG	This work
CBR008 (recP)	AAAGCGGTCGCGGTTAATCC	This work
SMD227 (nanA)	CATGGAGTTTAAGCCAGATG	This work
SMD228 (nanA)	GCCATAGTGAAGTACTCATC	This work

References

1. Tettelin, H.; Nelson, K.E.; Paulsen, I.T.; Eisen, J.A.; Read, T.D.; Peterson, S.; Heidelberg, J.; DeBoy, R.T.; Haft, D.H.; Dodson, R.J.; et al. Complete genome sequence of a virulent isolate of Streptococcus pneumoniae. *Science* **2001**, *293*, 498–506.
2. Domingues, S.; Aires, A.C.; Mohedano, M.L.; López, P.; Arraiano, C.M. A new tool for cloning and gene expression in Streptococcus pneumoniae. *Plasmid* **2013**, *70*, 247–253.
3. Moreira, R.N.; Domingues, S.; Viegas, S.C.; Amblar, M.; Arraiano, C.M. Synergies between RNA degradation and trans-translation in Streptococcus pneumoniae: cross regulation and co-transcription of RNase R and SmpB. *BMC Microbiol*. **2012**, 12, 268.
4. Bárria, C.; Domingues, S.; Arraiano, C.M. Arraiano, Pneumococcal RNase R globally impacts protein synthesis by regulating the amount of actively translating ribosomes. *RNA Biol.* **2019**, *16*, 211–219.
5. Simon, D.; Chopin, A. Construction of a vector plasmid family and its use for molecular cloning in Streptococcus lactis. *Biochimie* **1988**, *70*, 559–566.
6. Altincicek, B.; Vilcinskas, A. Metamorphosis and collagen-IV-fragments stimulate innate immune response in the greater wax moth, Galleria mellonella. *Dev. Comp. Immunol.* **2006**, *30*, 1108–1118.
7. Wojda, I.; Kowalski, P.; Jakubowicz, T. Humoral immune response of Galleria mellonella larvae after infection by Beauveria bassiana under optimal and heat-shock conditions. *J. Insect. Physiol.* **2009**, *55*, 525–531.
